# Inter-annual cascade effect on marine food web: A benthic pathway lagging nutrient supply to pelagic fish stock

**DOI:** 10.1371/journal.pone.0184512

**Published:** 2017-09-08

**Authors:** Lohengrin Dias de Almeida Fernandes, Eduardo Barros Fagundes Netto, Ricardo Coutinho, on behalf of the PELD-RECA

**Affiliations:** 1 Department of Biotechnology, Instituto de Estudos do Mar Almirante Paulo Moreira (IEAPM), Brazilian Navy, Arraial do Cabo, Rio de Janeiro, Brazil; 2 Marine Biotechnology Post-Graduation Program, Arraial do Cabo, Rio de Janeiro, Brazil; 3 Department of Oceanography, Instituto de Estudos do Mar Almirante Paulo Moreira (IEAPM), Brazilian Navy, Arraial do Cabo, Rio de Janeiro, Brazil; Universidade Federal do Rio de Janeiro, BRAZIL

## Abstract

Currently, spatial and temporal changes in nutrients availability, marine planktonic, and fish communities are best described on a shorter than inter-annual (seasonal) scale, primarily because the simultaneous year-to-year variations in physical, chemical, and biological parameters are very complex. The limited availability of time series datasets furnishing simultaneous evaluations of temperature, nutrients, plankton, and fish have limited our ability to describe and to predict variability related to short-term process, as species-specific phenology and environmental seasonality. In the present study, we combine a computational time series analysis on a 15-year (1995–2009) weekly-sampled time series (high-resolution long-term time series, 780 weeks) with an Autoregressive Distributed Lag Model to track non-seasonal changes in 10 potentially related parameters: sea surface temperature, nutrient concentrations (NO_2_, NO_3_, NH_4_ and PO_4_), phytoplankton biomass (as *in situ* chlorophyll *a* biomass), meroplankton (barnacle and mussel larvae), and fish abundance (*Mugil liza* and *Caranx latus*). Our data demonstrate for the first time that highly intense and frequent upwelling years initiate a huge energy flux that is not fully transmitted through classical size-structured food web by bottom-up stimulus but through additional ontogenetic steps. A delayed inter-annual sequential effect from phytoplankton up to top predators as carnivorous fishes is expected if most of energy is trapped into benthic filter feeding organisms and their larval forms. These sequential events can explain major changes in ecosystem food web that were not predicted in previous short-term models.

## Introduction

Globally, planktonic production and fish stocks are unevenly distributed in the oceans, particularly with respect to ecological boundaries, which are primarily defined in terms of temperature, salinity, light, and nutrient concentrations [1]. In view of the global importance of plankton production to fishery stocks [2,3], carbon cycling [3,4], and oxygen production, the analysis of inter-annual changes in ecological boundaries over decades can assist in more conscientious decisions regarding the conservation, management and exploitation of living resources in the oceans [5–7].

In past decades, a number of predictions concerning global changes in fishery stocks and plankton production have been based on large-scale coupled models that include physical, chemical and biological descriptors. The majority of these models have described large-scale spatial and short-scale temporal changes in plankton production and are highly dependent on indirect estimates of chlorophyll through variables such as water transparency, ocean color, temperature, and nutrient concentrations [8–10]. The scarcity of empirical observations to justify this approach has been reduced over the years as new time series become available [11–13], increasing the reliability of the predictions.

At a global scale, productivity in the South Atlantic Ocean is very low and primarily restricted to the Benguela System and the Brazil-Malvinas Confluence [14–16]. Excluding these and the coastal areas, primary production is strongly constrained by the presence of a quasi-permanent thermocline that maintains the oligotrophic condition of the photic layer. Curiously, such oligotrophic ecosystems, like most of the tropical southwestern Atlantic Ocean, are known to sustain highly-diverse pelagic and benthic communities. Based on this paradigm, any increase in biodiversity and/or productivity is related primarily to occasional disturbances in the vertical structure, such as wind-driven upwelling and mesoscale oceanic processes, which render the thermocline more shallow and transport new nutrients up from the deep layers [17]. In the bottom-up paradigm, this type of nutrient supply should suddenly increase the phytoplankton growth rate and thus the phytoplankton standing stock, whose energy and matter should then continue to flow through the food web, with a sequential cascade effect, up to the higher trophic levels such as the mesoplankton and small fishes [18–20]. This scenario occurs in the Cabo Frio region, known for the seasonal upwelling that boosts the energy transfer throughout the sequential cascade effect [21–23]. At a short time scale, from hours to a few days, upwelling nutrient inputs into the photic zone fuel the picoplankton and nanoplankton growth rates, which in turn promote the appearance of microplankton [24,25]. The upwelling in the Cabo Frio coastal region usually lasts for a few days, after which growth rates decrease to the background level. This event happens successively during spring and summer in this region, and its frequency is known to have high inter-annual variability [16]. On a seasonal scale, the effects of the upwelling on the phytoplankton standing stock are strongly dependent upon the frequency and intensity of the nutrient supply [11,26,27], mainly during the spring bloom. However, previous evidences in Cabo Frio region [11] reveal that in some years there are unexpected bottom-up effect that cannot be fully explained by seasonal upwelling. The effect of varying upwelling intensity and frequency is less well understood at the inter-annual scale, although a corresponding cascade effect up to the higher trophic levels, such as zooplankton and fishes, is expected to occur. To examine whether the bottom-up effect can be tracked over several years, we hypothesized that more frequent and intense upwelling periods generate over several years a sequential effect on the higher trophic levels in the oligotrophic subtropical Southwestern Atlantic Ocean.

## Materials and methods

Plankton samples have been collected weekly in triplicate since October 1994 at Cabo Frio Island, Arraial do Cabo, Brazil (23°S 042.01°W). Data from January 1995 to December 2009 (15 years) were included herein. All samples were taken in the IEAPM field test area in Cabo Frio Island under license number 54371–1, 30906–1, and 18460–1 (Sisbio/ICMBio/MMA).

*In situ* sea surface temperature (SST, °C) was measured at a depth of approximately 1 meter using a reversing thermometer mounted in a Nansen bottle. We used SST as a proxy of upwelling in the region because temperatures less than 17°C are related to the influences of South Atlantic Central Water (SACW) on the upper layer [28,29].

The sequential effect of bottom-up stimuli was tracked from the time of the appearance of cold, rich, newly upwelled waters through the micropelagic food web (nutrients, phytoplankton and meroplankton) to the planktivorous and carnivorous fishes. Nitrogen (nitrite, nitrate, and ammonia) and phosphorus (orthophosphate) concentrations (μM)–two limiting macronutrients for phytoplankton growth [30]–were estimated using spectrophotometry [31]. Water samples were taken from a depth of ± 1 meter using Nansen bottles and kept refrigerated onboard until laboratory analysis (<2 hours later). The phytoplankton biomass was indirectly estimated as the extracted chlorophyll *a* (mg m^-3^) from the same water samples. The meroplankton abundance (N m^-3^) was estimated from horizontal hauls (100 μm mesh size) and refers to all larvae found in the samples. Only meroplankton groups have been previously identified and counted, and thus there are no data for other planktonic groups. In Cabo Frio region, the meroplankton includes some highly abundant organisms, as barnacle and mussel larvae, that can be used as good indicators of whole plankton nourishment. The veliger larvae of Mytilidae (Mollusca, Bivalvia) and the nauplii of unidentified Cirripedia (Crustacea) were sorted and counted under different stereomicroscopes over the years. Other less frequent groups, such as the larvae of Annelida, Decapoda, and Echinodermata, were not included in the present study. All laboratory procedures were performed by the same two individuals throughout the experiment to keep consistency over the years. Monthly and annual fish anomalies were calculated from the total fish caught and the fish caught per unit effort (CPUE) in Arraial do Cabo from [32,33], and because the monthly catch for each fish species is unavailable for the region, we used the annual catch of Lebranche mullet (*Mugil liza* Valenciennes, 1836), Brazilian sardine (*Sardinella brasiliensis* (Steindachner, 1879)), and horse-eye jack (*Caranx latus* Agassiz, 1831) as estimates of changes in detritivorous, planktivorous, and carnivorous fishes, respectively. We do not account for the seasonal or annual changes in fish prices that can selectively affect total catch in the region over the time series. As an alternative, the monthly catch from July to June (of the following year) was integrated into the annual data to include the entire period of upwelling in the region and then reduce the price effects.

To evaluate the inter-annual changes in the time series, all data were converted to monthly anomalies. To reduce the seasonality and clarify the general inter-annual trend in the time series, the monthly anomalies were calculated for each month, considering the average and standard deviation for that specific month over the entire time series, and then smoothed with the moving-average 4253H algorithm [34]. In other words, each parameter in January 1995, for example, was normalized by the mean of all January values (1995–2009) and then divided by the standard deviation of all January values (1995–2009). The overall inter-annual trend in each time series from 1995 to 2009 was estimated using a linear regression model *Y* = *b* + *aX*, where the intercept ‘b’ is irrelevant, and the slope ‘a’ represents the monthly increase or decrease in values. The significance of the slopes was verified using Student´s t test. The relationships between SST, nutrients (NO_2_, NO_3_, PO_4_, and NH_4_), chlorophyll *a* (Chl *a*), meroplankton (total, Cirripedia, and Mytilidae), and fish abundance (both total catch and catch per unit effort—CPUE) were analyzed using time series analyses and cross-correlated. In addition to meroplankton as a whole, anomalies in Mytilidae and Cirripedia were correlated with the chlorophyll *a* time series. These two groups were chosen because they are frequent, abundant and easily recognized in the samples.

To address the potential inter-annual relationship between time series, the Spearman correlation coefficient (ρ) was calculated between the ranks of distances/dissimilarities in the annual average of: 1) environmental parameters (temperature and nutrients); 2) first trophic level (Chlorophyll *a*); and 3) second trophic level (Meroplankton). The Euclidian coefficient was set as measurement of distance between the annual average of environmental parameters (temperature and nitrogen) after normalization while the Bray-Curtis dissimilarities between years was computed for the annual average of chlorophyll *a* and meroplankton after standardization and log(x+1) transformation [35]. The same analysis was performed twice for meroplankton in order to address non-lagged (ρ) and one-year lagged (ρ2) cascade effect. The matrix of distances/dissimilarities were graphically summarized in a Multi-Dimensional Scaling ordination using the software PRIMER v6 [35]. The significance (p) of the actual correlations was confirmed after 999 rank permutations to reveal the proportion of virtual higher correlation values between time series. We do not account for lagged relationship between SST, nutrients, and Chlorophyll *a* that seems to be more immediate or seasonal [11].

To address the partial contribution of each variable in the relationship between time series, the Multivariate Granger causality was tested using the Autoregressive Distributed Lag (ADL) model:

$$y_{t}=a+\sum \beta_{i}x_{t-i}+v_{t},$$ (1)

which estimates the correlation coefficient (β) at each time lag (i) between the dependent ‘y’ and the explanatory ‘x’ variables. The correlation coefficient can be viewed as the slope of a linear regression, and in cases where β was statistically significant (β≠0), we rejected the null hypothesis that there is no Granger causality between those variables at time lag ‘i’ [36,37]. We limited the lag range in the model from 0, 12, and 24 months (respectively immediate, one year, and two years; β_0_, β_1_, and β_2_) for ‘x’ to avoid multicollinearity, which could lead to unreliable coefficient estimates. All coefficients were determined by means of the software StatSoft Statistica v8. Like the Spearman rank correlation, all lagged parameter were restricted to meroplankton. We have included the previous period effect (ρy_t-1_) and the residual error effect (v_t_) but excluded the intercept (*a*) because it has no biological meaning in our model.

$$y_{t}=\sum \beta_{1}x_{t-i}+\rho y_{t-1}+v_{t}$$ (2)

The overall fitting of the model was expressed as coefficient of determination (R^2^) and was estimated by partitioning the Regressive and the Residual Factor effects using one-way Analysis of Variance (ANOVA). The significance of the slope for each lag of ‘x’ was evaluated using a Student’s t-test. In cases with no stationary trend in the time series, as previously examined using linear regression, the data were transformed to the first differential. To test for persistent seasonality in the data after moving average smoothing, a cross-correlation analysis was performed on a monthly average matrix of 180 cases (= 15 years x 12 months) x 10 variables to distinguish between seasonal (<12 months) and non-seasonal (>12 months) significant lagged correlations. Previously, serial dependency of the data was checked through autocorrelation and partial autocorrelation functions.

Raw data can be accessed in the supplementary tables (S1–S8 Tables).

## Results

**Fig 1:**
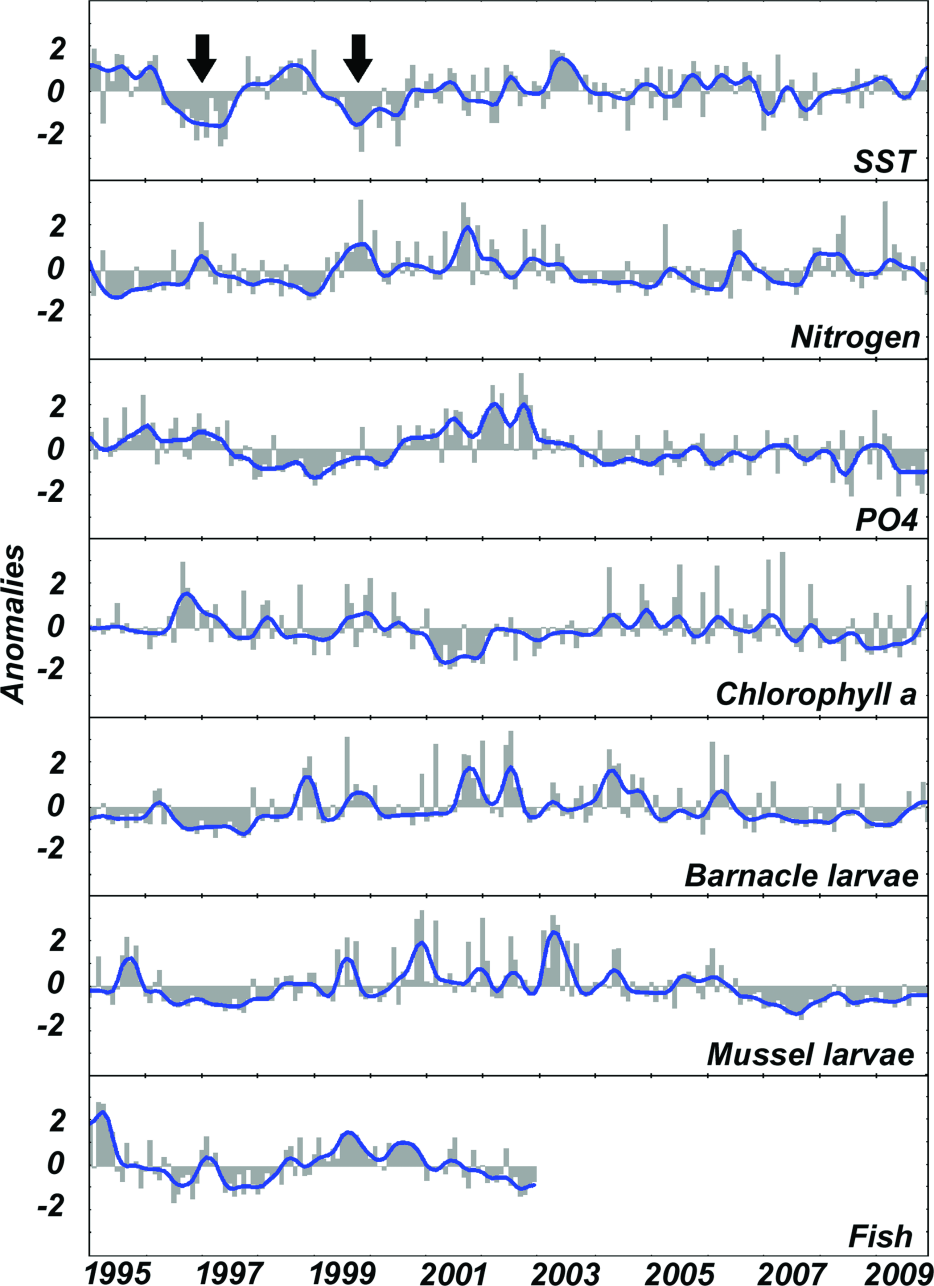
Smoothed month anomalies (blue line) of temperature (SST), nutrients (nitrite+nitrate and phosphate), phytoplankton (chlorophyll *a*), meroplankton (barnacle and mussel larvae), and fish (1995–2002 period available) superimposed on raw month anomalies (gray bars). The black arrows indicate major upwelling years.

Seasonally, SST in the area ranged between 15.9°C in the spring and 29.4°C in the autumn (Table 1). Strong negative anomalies that are indicative of upwelling were primarily observed during the summers of 1996–1997, 1999–2000, 2002 and 2007 (Fig 1, black arrows). Other years exhibited high internal variation with strong monthly changes (seasonal) instead of more frequent up- or downwelling.

**Table 1:** Descriptive statistics of the weekly raw data from January 1995 to December 2009 (~783 weeks).

	N	Mean	Min.	Max.	S.D.
**Temperature (°C)**	760	22.6	15.9	29.4	1.8
**NO**_**2**_ **(μM)**	775	0.08	0	1.00	0.09
**NO**_**3**_ **(μM)**	772	0.64	0	10.19	0.94
**NH**_**4**_ **(μM)**	775	1.30	0.07	7.85	0.83
**PO**_**4**_ **(μM)**	775	0.25	0	3.69	0.19
**Chlorophyll *a* (μM)**	740	0.89	0.01	6.28	0.59
**Mytilidae (N m**^**-3**^**)**	756	73	0	2636	146
**Cirripedia (N m**^**-3**^**)**	756	155	0	3641	288

No significant overall trend was found in sea surface temperature over the 15-years time series (Table 2). In contrast, significant inter-annual trend was revealed for nutrients as nitrite (+0.002 year^-1^ >> +0.0012 μM year^-1^), nitrate (0.004 year^-1^ >> +0.019 μM year^-1^), and phosphate (-0.005 μM year^-1^) in the region.

**Table 2:** Linear regression of the monthly anomalies (slope, r^2^, and p value).

	Slope	r^2^	p
**Temperature**	0.001	0.004	0.39
**(2000–03)**	0.03	0.21	0.001
**(2004–06)**	0.03	0.14	0.02
**(2007–09)**	0.03	0.18	0.01
**NO**_**2**_	0.002	0.02	0.04
**NO**_**3**_	0.004	0.04	0.008
**NH**_**4**_	0.001	0.002	0.59
**PO**_**4**_	-0.005	0.06	0.001
**Chlorophyll *a***	-0.0007	0.002	0.6
**Meroplankton**	-9.6E-5	0	0.94
**Mytilidae**	-0.002	0.01	0.15
**Cirripedia**	0.002	0.02	0.09
**Fish CPUE**	-0.004	0.02	0.20

Significant slopes (year^-1^) represent a single inter-annual trend of increase (positive) or decrease (negative) over the 15 years studied (1995–2009) or a partial inter-annual trend specifically for temperature (years).

A greater inter-annual change in temperature was observed from 1995 to 1999, including some of the highest (1995 and 1998) and lowest (1996 and 1999) anomalies in the time series. From 2000 to 2003, 2004 to 2006, and from 2007 to 2009, a partial linear trend for monthly SST anomalies exhibited positive slope, suggesting warming periods (approximately 0.16°C.year^-1^ 2000–2003; 0.02°C.year^-1^ 2004–2006; 0.08°C.year^-1^ 2007–2009) after an intense upwelling year.

A weak but significant negative correlation was found between SST and the total nitrogen (NO_2_+NO_3_+NH_4_) time series (r *=* -0.26; p *=* 0.04; n *=* 180), suggesting increases in nitrogen concentrations during upwelling. This correlation lagged by one month and becomes stronger when ammonia is removed (r *=* -0.47; p *=* 0.02; n *=* 180; Lag = 1 month). The strong positive anomalies in nitrogen that occurred in very cold years, such as 1997, 1999, 2001, and 2006 are primarily due to sudden increases in nitrite and nitrate concentrations during upwelling. This relationship is quite well predicted in our model (R^2^ = 0.67, p = 0.02) and virtually seasonal (β_0_ = -0.93, p = 0.01, Table 3) but somehow still dependent on nitrogen concentrations in the previous year (ρ = 0.64, p = 0.01).

**Table 3:** Parameter estimation of X (β) and Y (ρ) variables lagged from 0 to 2 years (respectively β_0_, β_1_, β_2_) by the autoregressive distributed lag analysis.

Independent	Dependent	Coefficients		t	p
**Temperature**	**NO**_**2**_**+ NO**_**3**_	β_0_	-0.93	-3.77	0.01*
		β_1_	-0.21	-0.06	0.32
		ρ	0.64	3.13	0.01*
**Temperature**	**PO**_**4**_	β_0_	-0.85	-1.85	0.10
		β_1_	-0.14	-0.15	0.68
		ρ	0.67	2.36	0.04*
**Temperature**	**Chl *a***	β_0_	0.16	0.60	0.56
		β_1_	0.03	0.12	0.90
		ρ	0.25	1.06	0.32
**NO**_**2**_**+ NO**_**3**_	**Chl *a***	β_0_	-0.28	-1.01	0.34
		β_1_	-0.12	-0.41	0.66
		ρ	-0.10	-0.26	0.80
**PO**_**4**_	**Chl *a***	β_0_	-0.24	-0.81	0.44
		β_1_	-0.08	-0.30	0.86
		ρ	0.10	0.36	0.73
**Chl *a***	**Cirripedia**	β_0_	0.06	0.20	0.85
		β_1_	-0.50	-1.88	0.09
		β_2_	0.13	0.40	0.70
		ρ	0.34	1.31	0.22
**Chl *a***	**Mytilidae**	β_0_	-0.34	-0.95	0.37
		β_1_	-0.15	-0.47	0.65
		β_2_	-0.36	-0.97	0.36
		ρ	0.40	1.52	0.16

*Significant (p<0.05) coefficients (β and ρ) in the model.

**Fig 2:**
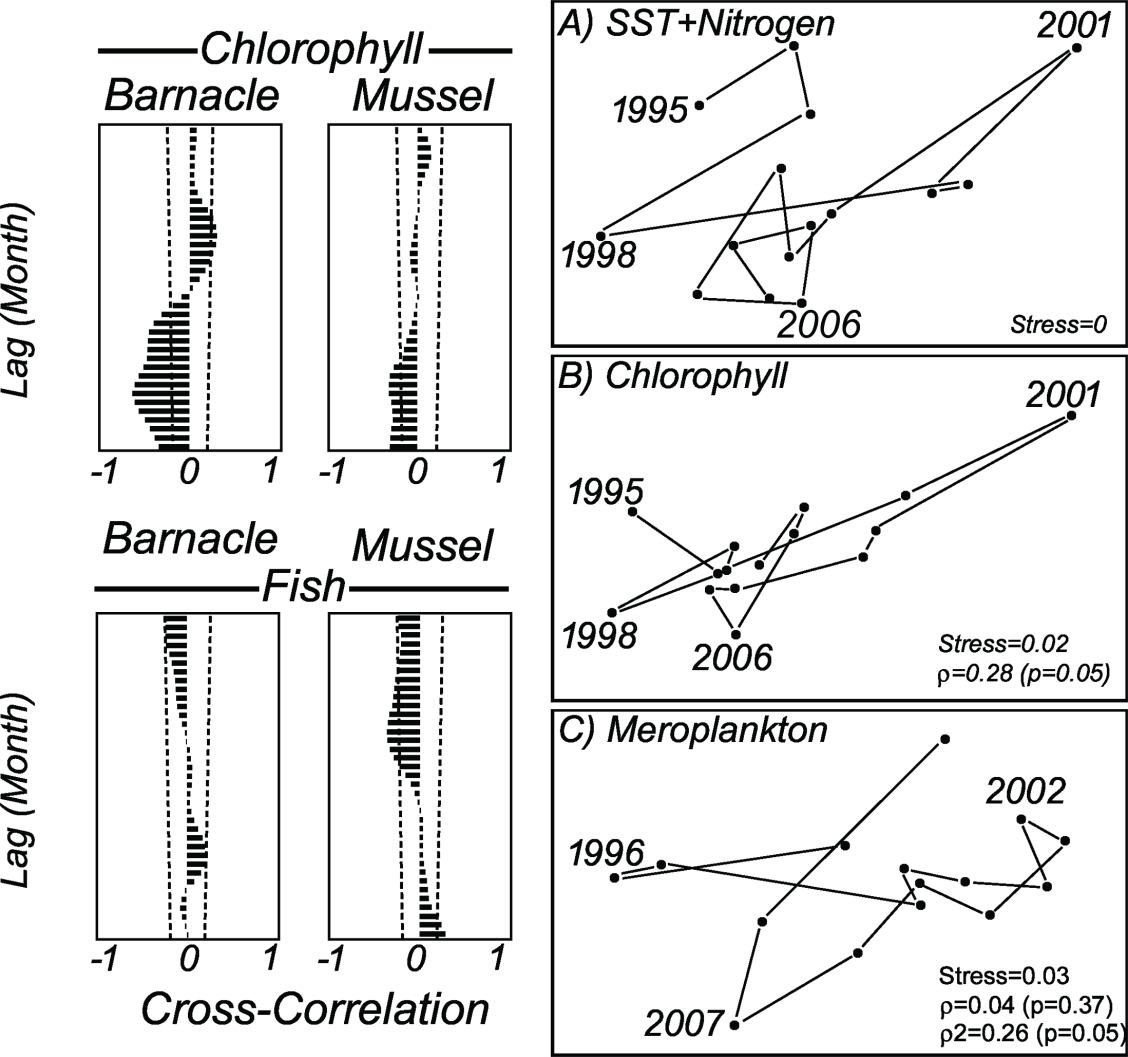
Correlation between major parameters time series and breakdown of temporal seriation. Left; Cross-correlogram (R) between chlorophyll *a*, meroplankton, and fish time series (N = 180 monthly smoothed anomalies). Top: chlorophyll *a* (non-lagged) and meroplankton (lagged); Bottom: meroplankton (non-lagged) and fish (lagged). The lag is in months. Right, Breakdown of seriation in inter-annual variation. Most important disruptions in (A) SST and nutrients, and (B) chlorophyll *a* trend that happen in 2001 and 2006 were followed by (C) meroplankton in the following years (2002/03 and 2007). Non-lagged Spearman rank correlation (ρ) and one-year lagged (ρ_2_) is shown inside the graph with p level in brackets.

These high-nitrogen/low-temperature years, particularly 2001, were evident as a breakdown of inter-annual seriation (Fig 2A). Most important disruptions in nutrient and chlorophyll (ρ = 0.28, p = 0.05) concentration occurred in 2001 and 2006 (Fig 2B) due to high nitrogen and low chlorophyll concentrations coincident with a high larval pool period in the following years (2002–2003) (Fig 2C). Among the most abundant meroplankton organisms in the region, the barnacle larvae exhibited higher inter-annual correlation with chlorophyll (Fig 2, left top) than mussel and fish (Fig 2, left bottom).

**Fig 3:**
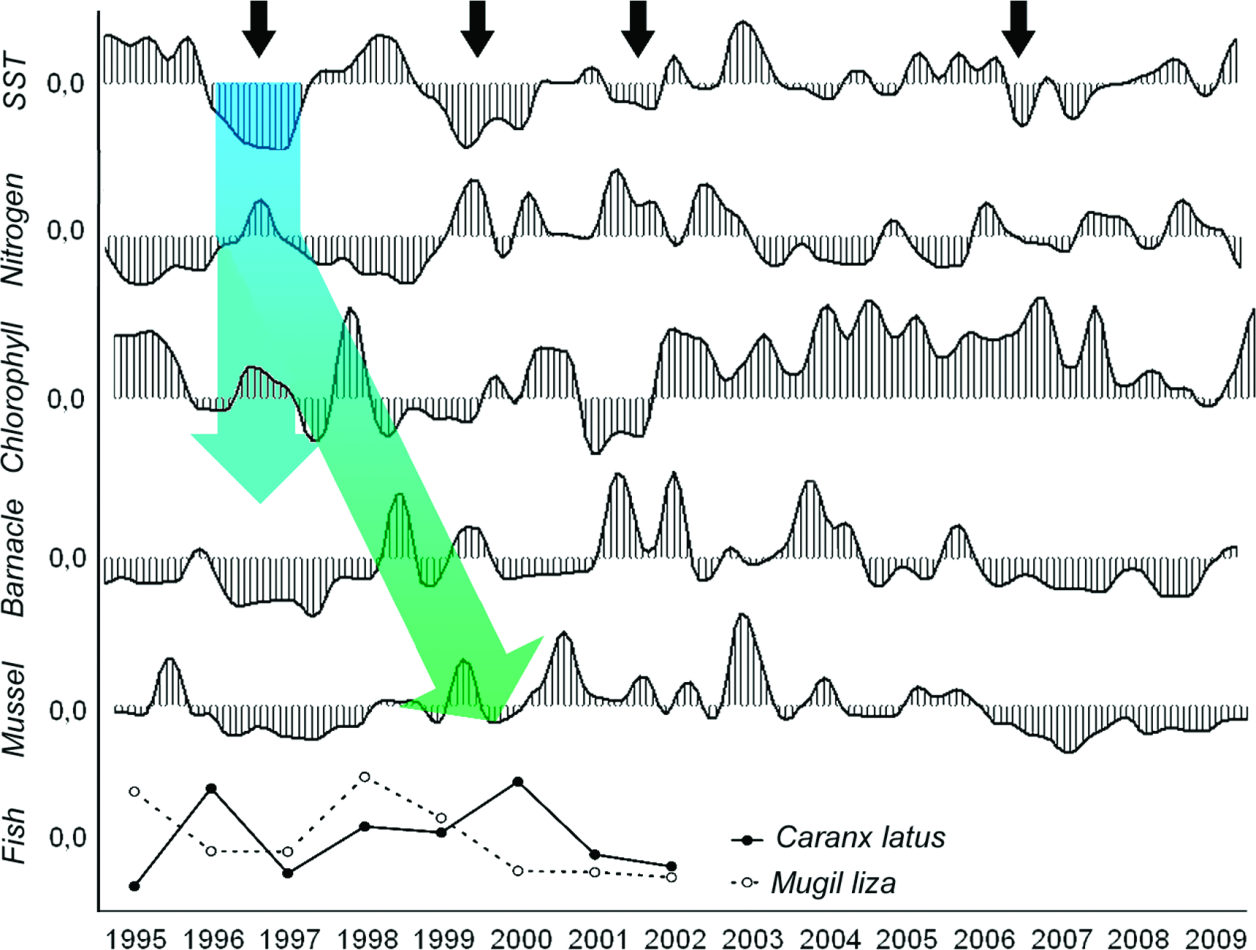
Inter-annual cascade effect on pelagic food web over the 15-years (1995–2009). Seasonal (vertical from temperature to phytoplankton) and inter-annual (oblique from chlorophyll to carnivorous fish) bottom-up stimuli that started in high frequent and intense upwelling years are transmitted to the next level in distinct pathways.

Our results reveal better fits between inter-annual changes in SST and nutrients than between nutrients, chlorophyll *a*, and meroplankton (Table 3). The abundance of meroplankton was negatively correlated with the inter-annual changes in chlorophyll *a*, with a persistent seasonal lag of six months for barnacle larvae (r *=* -0.64; p *=* 0.004; n *=* 180; Lag = 6 months) and seven months for mussel larvae (r *=* -0.33; p *=* 0.007; n *=* 180; Lag = 7 months). In addition, a significant cross-correlation was found between chlorophyll *a* and barnacle larvae, with a 12 month lag (r *=* -0.47; p *=* 0.006; n *=* 180; Lag = 12 months) due to a second annual peak in larval release. Because the most abundant larval groups, barnacles and mussel, alternate their annual peaks, usually every two years for cirripeds (1998/99, 2001/02, 2004) and each year for mussels (2000, 2003), no significant Spearman rank correlation was obtained when both groups are merged as meroplankton (ρ = 0.04, p = 0.37). If we focused exclusively on the relationship between barnacle nauplii and chlorophyll *a* over the years, time series get more closely related when larval abundance is lagged in one year. This lagged relationship was suggested by the strong increase in the correlation coefficient from β_0_ = 0.06 (p = 0.85) to β_1_ = -0.50 (p = 0.09), even that still not significant. Additional evidences that inter-annual changes in the larval pool are following that of chlorophyll *a* but lagged by one year (Fig 3) are found in the spatial distribution of years in the Multidimensional Scaling analyses (ρ_2_ = 0.26, p = 0.05, Fig 2B and 2C). From 1995 to 2001, the strongest inter-annual differences were evident for all parameters. After 2001, the spatial ordination of the annual average for environmental parameters (temperature and nitrogen) and chlorophyll *a* grouped the years closer than that for meroplankton, whose inter-annual variability remains high.

**Fig 4:**
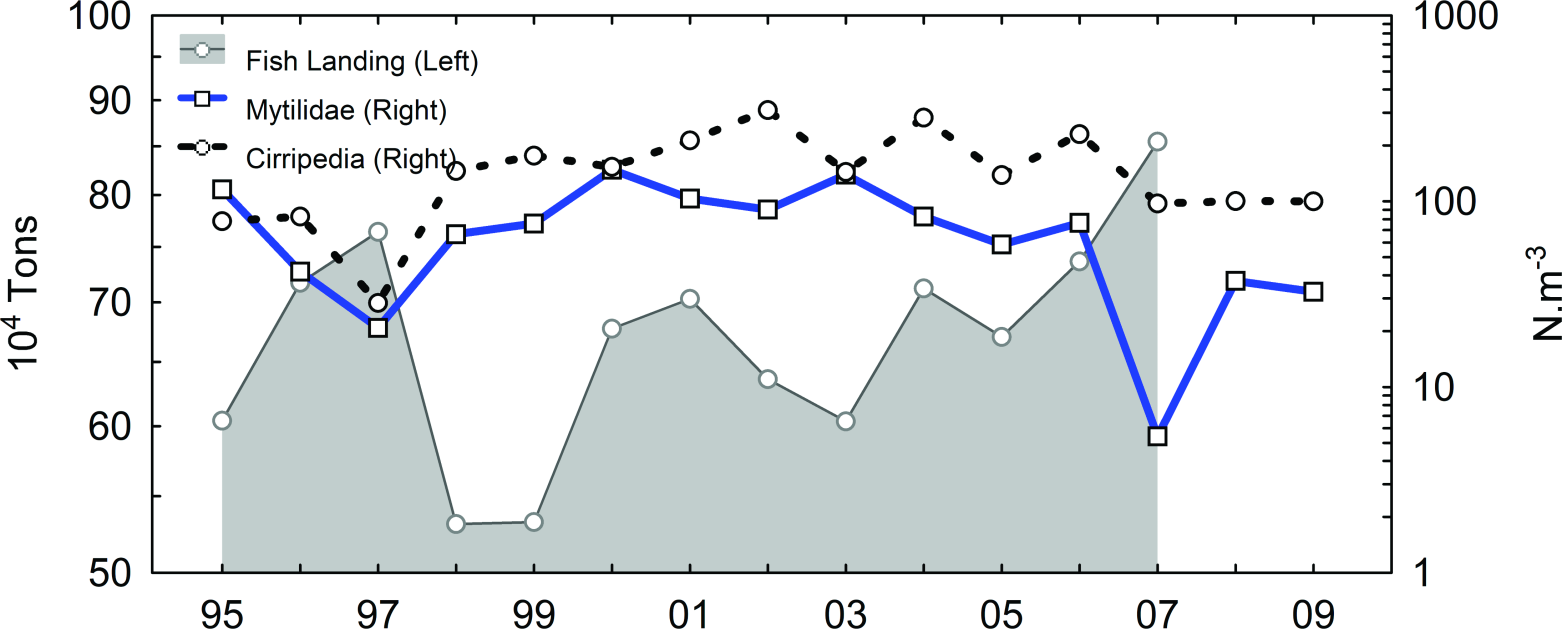
Annual average of barnacle nauplii (Cirripedia; black dotted line) and mussel larvae (Mytilidae; blue continuous line) superimposed on total fish catch (grey area). Fishery data derived from [32,33].

A significant positive inter-annual correlation was found between the larval pool and sea temperature, with a one-year lag for barnacle larvae (r *=* 0.27; p *=* 0.007; n *=* 180; Lag = 12 months) and no lag for mussel larvae (r *=* 0.30; p *=* 0.006; n *=* 180; Lag = 0). As a whole, the meroplankton time series exhibits an increase in the larval pool after 1997 for barnacles and mussels. These increases follows the major disruption in the fish catch that began after 1997 (Fig 4). Inter-annual changes in fish abundance in the Cabo Frio region occurred over approximately half of the 95–09 period; as in the case of meroplankton, which includes different groups with distinct feeding habits, no significant correlation was evident until the dominant groups were split. Positive anomalies in the number of fish caught were observed through 1996 and 1997, after which anomalies were reduced to less than the overall average.

Examining only two common planktivorous and carnivorous fish species in the region, peaks in the time series coincided with (Fig 3, peaks in *Mugil liza*) or immediately followed (Fig 3, peaks in *Caranx latus*) major peaks in phytoplankton in 1995 and 1998. The clearest relationship between inter-annual changes in the larval pool and fish abundance was observed in 1997 and 2007, when the annual decrease in larval release coincided with a peak in the total fish catch (Fig 4).

## Discussion

### Initial bottom-up effects of upwelling on nutrients and phytoplankton

In contrast with previous studies conducted in the same region from 1972 to 1978, which found monthly average SSTs of 16.0°C to 24.1°C [38], the current study found that the seasonal differences in temperature between winter and summer increased over the years, from 15.9°C in the spring up to 29.4°C in the autumn. We consider that there were no differences in the lowest temperatures, but a significant warming. Similarly, changes in nutrient concentrations are markedly seasonal in the Cabo Frio region [11], where both wind-induced and meander-induced upwelling are thought to cause thermocline to become more shallow and to bring deep nutrient-rich waters to the photic layer [39]. The inter-annual change in temperature and nutrients lead us to hypothesize that any increase in the frequency and intensity of upwelling can alter the ecosystem physiology and the budget of nutrients available to phytoplankton. Based on the data presented in this study, more frequent and strong upwelling events, such as those observed in 1996/97 and 1999/2000, resulted in an almost immediate bottom-up sequential effect through nutrient inputs into the photic zone. These inputs were revealed by the significant negative correlation between new nitrogen (nitrite and nitrate) and temperature due to the increase in nitrogen concentrations during upwelling. The subsequent effects ranged from immediate (vertical; lag = 0) as previously revealed [11] to delayed (long-term or oblique; >1 year lag) according to the pathway that energy flows in the food web. Examples of immediate response were seen in SST, nutrients, and chlorophyll a correlations (Fig 2A and 2B). Considering the potential confounding effects of temperature and nutrients on marine communities [40], the differences in the nutrient composition over the years were well correlated with the occurrence of strong upwelling, suggesting that temperature change initiates the bottom-up cascading effects on the food web. The response of the nutrient concentrations to any increase in upwelling intensity was mostly vertical as no lagged response was found in our model. In contrast, the time response for the next steps in the energy flow (plankton and fishes) varied from immediate to a one-year lag. In the present study, vertical bottom-up transference was suggested from nutrients to phytoplankton and to some planktivorous/detritivorous fishes, such as *Mugil liza*, that benefit from this vertical flow through a relatively small number of trophic levels. In this particular case, the young and adult stages may have access to the newly available energy to grow and reproduce. In contrast, those organisms that depend on additional transfers of nutrients across a greater number of trophic levels throughout their growth and development are likely to exhibit a lagged and less intense response. For example, non-reproductive barnacles and mussels that benefits from spring bloom can spend the majority of this new input of energy in growth and/or gonadal development that in turn will result in an increased larval pool in the next year. This delayed response is mostly dependent on the ontogenetic development (S1 Fig). Unstructured pelagic food webs and convoluted energy flows in the plankton were suggested to be related to ontogenetic development [41].

Within certain limits, temporal and spatial changes in plankton productivity appear to be more strongly related to the degree of complexity in the size-structured food web [42,43]. In contrast with the vertical bottom-up stimulus and its resulting cascade effect, which usually lasts for weeks or a few months, oblique bottom-up transfers of energy and matter can extend over several years [5,44,45].

The high concentrations of nutrients that coincided with very cold years in the time series suggest that both the frequency and the intensity of upwelling events can play a major role in the inter-annual sequential effect. Sudden changes in both parameters–frequency and intensity–in 1997/98, 2001, and 2006 resulted in the breakdown of the temporal seriation of upwelling that in turn affected the phytoplankton and meroplankton seasonality in the following years. The strong breakdown revealed in 2001 and 2006 for SST and chlorophyll *a*, and in the next years (2002 and 2007) for meroplankton was coincident with the change in upwelling rhythm. Despite natural eutrophication, this region typically presents moderate primary productivity with seasonal blooms of short duration and low intensity [23,46]. Similarly, although cold waters are associated with increasing nutrient concentrations, these waters can also slow the metabolism of organisms and reduce consumption by phytoplankton, preventing the rapid depletion of nutrients. Although seasonal depletion of nutrients via phytoplankton intake can return the environment to an oligotrophic state [11,47], additional inputs of nitrogen over months and years continue to fuel phytoplankton growth on a longer-than-seasonal scale. These inter-annual cascading changes in chlorophyll *a*, barnacle, and mussel larvae were well predicted in our autoregressive model when the changes in temperature, nitrogen, and phytoplankton in the previous year were included. Similarly, after the removal of the immediate response of phytoplankton to eutrophication, the significant cross-correlation (one-year lag) of nitrogen, chlorophyll *a*, and barnacle larvae supports the hypothesis that the bottom-up stimuli are transferred to higher trophic levels over the years. In this sense, a large proportion of this new nitrogen that was incorporated into the food web after strong upwelling periods becomes available again to the size-structured pelagic food web in the next year. As an example of such complexity in the nutrient-phytoplankton relationship, some peaks in the chlorophyll *a* time series that occur in warm, oligotrophic years were uncoupled from upwelling and are most likely due to the increased recycling of nutrients and/or reduced grazing pressure. These ´unexpected´ peaks in chlorophyll *a* were followed in the subsequent months by a sudden increase in the larval pool, as in 1998, suggesting a top-down control that prevents the continued growth of phytoplankton. A high ammonia concentration during these years is indicative of increased recycling through the microbial loop. Under certain conditions, picoplankton can contribute most of the production in Cabo Frio [48,49], fuelling nitrogen consumption and increasing the ammonia concentration.

### Inter-annual bottom-up effects of phytoplankton on higher trophic levels

As a whole, the meroplankton comprises organisms with distinct trophic niches, and the interspecific relationships of these organisms are too highly complex to be directly correlated with phytoplankton. For example, barnacles and mussels release their larvae at different lag times relative to the seasonal spring bloom of phytoplankton [11]. This lagged cascading effect explains the more reliable correlations that appeared when more abundant groups were split in the analysis. Certain highly abundant groups in the meroplankton of the Cabo Frio region, such as Cirripedia and Mytilidae [50], are well known to exhibit a positive correlation between the parental development of gonads and the chlorophyll *a* concentration [50–52]. These filter-feeder organisms can retain food particles over a wide size range that includes small phytoplankton cells [53,54], sometimes exerting a highly efficient top-down control on phytoplankton [55] and likely leading to better nourishment and greater fecundity. Such top-down control cannot be measured as an immediate sequential effect, as larval abundance can, because there is a delay between adult nourishment and larval release. Results from our model reveal a significant inter-annual correlation lagged by ~1 to 2 years that supports deviation from the classical bottom-up stimulus, as expected in case of parental development of gonads, increasing offspring only in the next years. We hypothesized that top-down control exerted by non-reproductive stages can lead to oblique (or inter-annual) sequential cascading effect in the food web.

Two species of Mytilidae, *Brachidontes solisianus* and *Perna perna* [56], are dominant at the study site; there are more species of Cirripedia. Comparatively, Mytilidae includes fewer species than Cirripedia in the region, and the mussel *Perna perna* is one of the most abundant Mytilidae in the region [57]. This species has a high growth rate (24–27 mm.year^-1^) relative to other Mytilidae, and although some individuals start to reproduce early in their development, the majority (approximately 90% of the population) reach fertility only after the first year [58]. Strong pulses of the Mytilidae larval pool that occurred 12–18 months after the first increase in phytoplankton biomass in 1998 and 2000/01 were coincident with the expected time for a combined bottom-up sequential effect through food web and ontogenetic development (S1 Fig). For Cirripedia, the adults of *Tetraclita* sp., a highly abundant genus in the Cabo Frio region, can rest for one year, actively feeding upon phytoplankton and allocating a greater proportion of energy to the development of eggs and larvae instead of to growth [59]. In addition to the seasonal spring bloom of phytoplankton (vertical response), frequent and intense upwelling years that increase the phytoplankton standing stock may also lead to increased adult fecundity, that in turns starts an inter-annual bottom-up sequential effect.

Similarly to barnacle and mussel larvae, inter-annual changes in fish recruitment can be correlated with the frequency and intensity of upwelling [60]. Considering only the herbivorous Lebranche mullet (*Mugil liza*) [61,62], peaks in abundance in 1995 and 1998 coincided with high *in situ* chlorophyll *a* concentration, and like other Mugilidae that also consume diatoms, *M*. *liza* is expected to vary in abundance directly (vertical relationship) with the inter-annual variation in phytoplankton biomass. Coincident peaks in the annual abundance of *Mugil liza* and phytoplankton in 1995 and 1998 provide evidence of such a relationship. According to Rueda[63], stomach content analysis of two Mugilidae species revealed detritus and benthic diatoms as the dominant food items. More recent studies of diet composition and seasonal feeding variation in *Mugil cephalus* found mud, diatoms and green algae as the primary diet, and blue-green algae and dinoflagellates as secondary [64,65]. In contrast, the abundance of *Caranx latus* lagged relative to phytoplankton, and the two major peaks in 1996 and 2000 appear to be correlated with Mytilidae, with a one-year lag. If inter-annual changes in the nutrient budgets can be transmitted over the years, we expect different trophic levels to exhibit similar but delayed time series trends. Thus, both the *Caranx latus* and Mytilidae time series can behave similarly but with distinct lags in response to the inter-annual growth of phytoplankton. No lag was observed between phytoplankton biomass and annual fish production in the region, and the high correlation furnishes strong evidence of the fast bottom-up effect. Similar in economic importance to the herbivorous Lebranche mullet, the family Clupeidae includes one of the most important species of fish caught in the region: the planktivorous Brazilian sardine (*Sardinella brasiliensis*) [66–68]. Both planktivorous Mugilidae and Clupeidae support up to 50% of the total fishery in the region [33].

Top-down control is usually opposed to the bottom-up effect because the more efficient vertical control exerted by one level results in a less evident sequential stimulus through the food web. Several studies have argued that the spring bloom of phytoplankton does not generate a sequential effect if the consumption by microplankton keeps the growth of the phytoplankton below certain limits [69,70]. Simultaneous bottom-up and top-down effects with multiples switches, from copepods to gelatinous plankton, are thought to control sequential effects through the food web to higher trophic levels [71,72]. To generate a sequential effect, the phytoplankton growth rate should be high enough to prevent immediate top-down and lateral control. As previously stated, the resulting interaction between the bottom-up and top-down effects is time-scale dependent [44], and according to our results, it is also frequency and intensity dependent.

Although certain bottom-up effects apparent in our data could be biased by the large number of variables included in the analysis, our results suggest that strong inter-annual changes in SST can be followed by an increase in fish abundance through effects on nutrients and phytoplankton and sometimes through the additional levels such as meroplankton. The mechanism by which long-term changes in abiotic conditions can impact species interactions and cause inter-annual changes in the relative importance of bottom-up and top-down control upon primary production can thus be better understood [44]. Apparently, the more levels in the size-structured food web, the more oblique (inter-annual) the sequential effect. Based on several similar relationships, sequential effects have already been revealed between phytoplankton and the living resources at the study site. These resource organisms include squid [73], Brazilian sardine, Argentine anchovy [16,74], and even birds such as terns [75]. In this sense, highly productive years that result from intense and frequent upwelling can promote the growth of fish populations with up to a one-year lag. By including this effect in the new models, we expect to better predict changes in the long temporal-scale events in major marine ecosystems in the Southwestern Atlantic Ocean.

## Supporting information

S1 Table Monthly anomalies of SST. (DOCX)

S2 Table Monthly anomalies of NO2+NO3. (DOCX)

S3 Table Monthly anomalies of phosphate. (DOCX)

S4 Table Monthly anomalies of chlorophyll *a*. (DOCX)

S5 Table Monthly anomalies of barnacle larvae (exclusively nauplii). (DOCX)

S6 Table Monthly anomalies of mussel larvae (exclusively Mytilidae). (DOCX)

S7 Table Monthly anomalies of fish. (DOCX)

S8 Table Annual anomalies of fish. (DOCX)

S1 Fig Conceptual model of interference in the flux of energy throughout the classical size-structured food web. Energy and matter consumed by phytoplankton started a bottom-up stimulus that is not completely transmitted to the next trophic level in the plankton. Benthic filter-feeding organisms like barnacles and mussels can consume the majority of phytoplankton (Filtration) exerting a competitive top-down control that will start an inter-annual cascading effect. Settlement is an additional process that can trap some energy into the ontogenetic development of benthic organisms and alter the energy flow in the planktonic food web.
(TIF)

## References

[1] Mousing E, Ellegaard M, Richardson K. Global patterns in phytoplankton community size structure—evidence for a direct temperature effect. Mar Ecol Prog Ser. 2014;497: 25–38. doi: 10.3354/meps10583

[2] Beaugrand G, Kirby RR. Climate, plankton and cod. Glob Chang Biol. 2010;16: 1268–1280. doi: 10.1111/j.1365-2486.2009.02063.x

[3] Beaugrand G, Brander KM, Lindley JA, Souissi S, Reid PC. Plankton effect on cod recruitment in the North Sea. Nature. 2003;426: 661–664. doi: 10.1038/nature02164 14668864 10.1038/nature02164

[4] Durant JM, Hjermann DØ, Ottersen G, Stenseth NC. Climate and the match or mismatch between predator requirements and resource availability. Clim Res. 2007;33: 271–283.

[5] Halpern BS, Cottenie K, Broitman BR. Strong top-down control in southern California kelp forest ecosystems. Science. 2006;312: 1230–1232. doi: 10.1126/science.1128613 16728644 10.1126/science.1128613

[6] Ji R, Edwards M, Mackas DL, Runge JA, Thomas AC. Marine plankton phenology and life history in a changing climate: current research and future directions. J Plankton Res. 2010;32: 1355–1368. doi: 10.1093/plankt/fbq062 20824042 10.1093/plankt/fbq062 PMC2933132

[7] Straile D, Stenseth N. The North Atlantic Oscillation and ecology: links between historical time-series, and lessons regarding future climate warming. Clim Res. 2007;34: 259–262. doi: 10.3354/cr00702

[8] Geider RJ, Delucia EH, Falkowski PG, Finzi AC, Grime JP, Grace J, et al Primary productivity of planet earth: biological determinants and physical constraints in terrestrial and aquatic habitats. Glob Chang Biol. 2001;7: 849–882.

[9] Boyce DG, Lewis MR, Worm B. Global phytoplankton decline over the past century. Nature. 2010;466: 591–596. doi: 10.1038/nature09268 20671703 10.1038/nature09268

[10] Skogen MD, Budgell WP, Rey F. Interannual variability in Nordic seas primary production. ICES J Mar Sci. 2007;64: 889–898.

[11] Fernandes LD de A, Quintanilha J, Monteiro-ribas W, Gonzalez-Rodriguez E, Coutinho R. Seasonal and interannual coupling between sea surface temperature, phytoplankton and meroplankton in the subtropical south-western Atlantic Ocean. J Plankton Res. 2012;34: 236–244. doi: 10.1093/plankt/fbr106

[12] Highfield J, Eloire D, Conway DVP, Lindeque PK, Attrill MJ, Somerfield PJ. Seasonal dynamics of meroplankton assemblages at station L4. J Plankton Res. 2010;32: 681–691. doi: 10.1093/plankt/fbp139

[13] Frank KT, Petrie B, Fisher JAD, Leggett WC. Transient dynamics of an altered large marine ecosystem. Nature. 2011;477: 86–89. doi: 10.1038/nature10285 21796120 10.1038/nature10285

[14] Le Moigne FAC, Boye M, Masson A, Corvaisier R, Grossteffan E, Guéneugues A, et al Description of the biogeochemical features of the subtropical southeastern Atlantic and the Southern Ocean south of South Africa during the austral summer of the International Polar Year. Biogeosciences. 2013;10: 281–295. doi: 10.5194/bg-10-281-2013

[15] Field CB, Behrenfeld MJ, Randerson JT, Falkowski P. Primary Production of the Biosphere: Integrating Terrestrial and Oceanic Components. Science. 1988;281: 237–240. doi: 10.1126/science.281.5374.237 10.1126/science.281.5374.237 9657713

[16] Coelho-Souza SA, López MS, Guimarães JRD, Coutinho R, Candella RN. Biophysical interactions in the Cabo Frio upwelling system, southeastern Brazil. Brazilian J Oceanogr. 2012;60: 353–365.

[17] Levinton JS. Critical Factors in Plankton Abundance Processes in the Open Sea. 4th ed. Oxford University Press; 2013 pp. 225–257.

[18] Chassot E, Bonhommeau S, Dulvy NK, Watson R, Gascuel D, Pape O Le. Global marine primary production constrains fisheries catches. Ecol Lett. 2010;13: 495–505. doi: 10.1111/j.1461-0248.2010.01443.x 20141525 10.1111/j.1461-0248.2010.01443.x

[19] Edwards M, Richardson AJ. Impact of climate change on marine pelagic phenology and trophic mismatch. Nature. 2004;430: 881–884. doi: 10.1038/nature02808 15318219 10.1038/nature02808

[20] Landry MR, Calbet A. Microzooplankton production in the oceans. J Mar Sci. 2004;61: 501–507. doi: 10.1016/j.icesjms.2004.03.011

[21] Carbonel CAA, Valentin JL. Numerical modelling of phytoplankton bloom in the upwelling ecosystem of Cabo Frio (Brazil). Ecol Modell. 1999;116: 135–148.

[22] Valentin JL. Analyse des paramètres hydrobiologiques dans la remontée de Cabo Frio (Brésil). Mar Biol. 1984;82: 259–276.

[23] Valentin JL, Lins da Silva NM. Le plancton dans l’upwelling de Cabo Frio (Brésil): microrépartition spatio-temporelle à une station fixe. Ann da L’Institut Océanographique. 1986;62: 117–135.

[24] Guenther M, Gonzalez-Rodriguez E, Carvalho WF, Rezende CE, Mugrabe G, Valentin JL. Plankton trophic structure and particulate organic carbon production during a coastal downwelling-upwelling cycle. Mar Ecol Prog Ser. 2008;363: 109–119. doi: 10.3354/meps07458

[25] McManus GB, Costas BA, Dam HG, Lopes RM, Gaeta SA, Susini SM, et al Microzooplankton grazing of phytoplankton in a tropical upwelling region. Hydrobiologia. 2006;575: 69–81. doi: 10.1007/s10750-006-0279-9

[26] Corbisier T, Petti M, Soares L, Muto E, Bromberg S, Valiela I. Trophic structure of benthic communities in the Cabo Frio upwelling system (southeastern Brazilian shelf): a temporal study using stable isotope analysis. Mar Ecol Prog Ser. 2014;512: 23–38. doi: 10.3354/meps10947

[27] Soares L, Muto E, Lopez J, Clauzet G, Valiela I. Seasonal variability of δ13C and δ15N of fish and squid in the Cabo Frio upwelling system of the southwestern Atlantic. Mar Ecol Prog Ser. 2014;512: 9–21. doi: 10.3354/meps10948

[28] Mémery L, Arhan M, Alvarez-Salgado XA, Messias M-J, Mercier H, Castro CG, et al The water masses along the western boundary of the south and equatorial Atlantic. Prog Oceanogr. 2000;47: 69–98. doi: 10.1016/S0079-6611(00)00032-X

[29] Saldanha-Corrêa FMP, Gianesella SMF. A microcosm approach on the potential effects of the vertical mixing of water masses over the primary productivity and phytoplankton biomass in the Southern Brazilian coastal region. Brazilian J Oceanogr. 2004;52: 167–182.

[30] Smith S V. Phosphorus versus nitrogen limitation in the marine environment. Limnol Oceanogr. 1984;29: 1149–1160.

[31] Parsons TR, Maita Y, Lalli CM. A manual of chemical and biological methods for seawater analysis 1st ed. New York: Pergamon Press; 1984.

[32] Silva P de A. Onze anos de produção pesqueira na região de Arraial do Cabo–RJ. Universidade Federal Fluminense 2004.

[33] CGFAP/DBFLO/IBAMA. Estatística da pesca 2007. Brasil: Grandes Regiões e Unidades da Federação Brasilia; 2007.

[34] Velleman PF. Definition and comparison of robust nonlinear data smoothing algorithms. J Am Stat Assoc. 1980;75: 609–615.

[35] Clarke KR, Warwick RM. Change in Marine Communities: An approach to Statistical Analysis and Interpretation. Plymouth: Primer-E: Plymouth; 2001.

[36] Pesaran MH, Shin Y. An Autoregressive Distributed Lag Modelling Approach to Cointegration Analysis. Econom Soc Monogr. 1998;31: 371–413.

[37] Hamilton JD. Time Series Analysis. Princeton University Press; 1994.

[38] Valentin JL. Spatial structure of the zooplankton community in the Cabo Frio region (Brazil) influenced by coastal upwelling. Hydrobiologia. 1984;113: 183–199.

[39] Acha EM, Mianzan HW, Guerrero RA, Favero M, Bava J. Marine fronts at the continental shelves of austral South America: Physical and ecological processes. J Mar Syst. 2004;44: 83–105. doi: 10.1016/j.jmarsys.2003.09.005

[40] López-Urrutia Á, Morán XAG. Temperature affects the size-structure of phytoplankton communities in the ocean. Limnol Oceanogr. 2015;60: 733–738. doi: 10.1002/lno.10049

[41] Steinberg DK, Landry MR. Zooplankton and the Ocean Carbon Cycle. Ann Rev Mar Sci. 2017;9: 413–444. doi: 10.1146/annurev-marine-010814-015924 27814033 10.1146/annurev-marine-010814-015924

[42] Manríquez K, Escribano R, Hidalgo P. The influence of coastal upwelling on the mesozooplankton community structure in the coastal zone off Central/Southern Chile as assessed by automated image analysis. J Plankton Res. 2009;31: 1075–1088.

[43] Behrenfeld MJ, Boss ES. Resurrecting the ecological underpinnings of ocean plankton blooms. Ann Rev Mar Sci. 2014;6: 167–194. doi: 10.1146/annurev-marine-052913-021325 24079309 10.1146/annurev-marine-052913-021325

[44] Kerimoglu O, Straile D, Peeters F. Seasonal, inter-annual and long term variation in top–down versus bottom–up regulation of primary production. Oikos. 2013;122: 223–234.

[45] Horn H, Horn W. Bottom-up or top-down–How is the autotrophic picoplankton mainly controlled? Results of long-term investigations from two drinking water reservoirs of different trophic state. Limnologica. 2008;38: 302–312. doi: 10.1016/j.limno.2008.05.007

[46] Valentin JL, Coutinho R. Modelling maximum chlorophyll in the Cabo Frio (Brazil) upwelling: a preliminary approach. Ecol Modell. 1990;52: 103–113. doi: 10.1016/0304-3800(90)90011-5

[47] Gonzalez-Rodriguez E. Yearly variation in primary productivity of marine phytoplankton from Cabo Frio (RJ, Brazil) region. Hydrobiologia. 1994;294: 145–156.

[48] Carvalho WF, Gonzalez E. Development of primary and bacterial productivity in upwelling waters of Arraial do Cabo region, RJ (Brazil). Brazilian J Oceanogr. 2004;52: 35–45.

[49] Guenther M, Valentin JL. Bacterial and phytoplankton production in two coastal systems influenced by distinct eutrophication processes. Oecologia Bras. 2008;12: 172–178.

[50] Skinner LF, Kelen H, Macharet L, Coutinho R. Influence of upwelling and tropical environments on the breeding development of the intertidal barnacle Tetraclita stalactifera (Lamarck, 1818). Brazilian J Oceanogr. 2011;59: 349–356.

[51] Darriba S, Sanjuan F, Guerra A. Energy storage and utilization in relation to the reproductive cycle in the razor clam (Jeffreys, 1865). ICES J Mar Sci. 2005;62: 886–896. doi: 10.1016/j.icesjms.2005.02.010

[52] Desai D, Anil A, Venkat K. Reproduction in Balanus amphitrite Darwin (Cirripedia: Thoracica): influence of temperaure and food concentration. Mar Biol. 2006;149: 1431–1441.

[53] Fréchette M, Butman CA, Rockwell Geyer W. The importance of boundary-layer flows in supplying phytoplankton to the benthic suspension feeder, Mytilus edulis L. Limnol Oceanogr. 1989;34: 19–36.

[54] Cibic T, Blasutto O, Bettoso N. Microalgal–meiofaunal interactions in a sublittoral site of the Gulf of Trieste (northern Adriatic Sea, Italy): A three-year study. J Exp Mar Bio Ecol. 2009;370: 144–154. doi: 10.1016/j.jembe.2008.12.006

[55] Cloern JE. Does the Benthos Control Phytoplankton Biomass in South San Francisco Bay? Mar Ecol Prog Ser. 1982;9: 191–202.

[56] Monteiro-Ribas W, Rocha-Miranda F, Romano RC, Quintanilha J. Larval development of Brachydontes solisianus (Bivalvia, Mytilidae), with notes on differences between its hinge system and that of the Mollusk Perna perna. Brazilian J Biol. 2006;66: 109–116. 10.1590/s1519-69842006000100014 16680313

[57] Rapagnã L. Estudo da estrutura das populações dos Bivalves Isognomon bicolor (C. B. Adams, 1845), Perna perna (Linnaeus, 1758) e Pinctada imbricata (Röding, 1798) nos Costões Rochosos de Arraial do Cabo, RJ, Brasil. Universidade Federal Fluminense 2004.

[58] Marques H, Ferreira J, Gelli V, De Moraes R, Nalesso R, Marenzi A. Biologia e Ecologia de Adultos In: Resgalla C Jr, Weber L, Da Conceição M, editors. O Mexilhão Perna perna (L): biologia, ecologia e aplicações. Rio de Janeiro: Interciência; 2008 pp. 55–68.

[59] Villalobos CR. Variations in population structure in the genus Tetraclita (Crustacea: Cirripedia) between temperate and tropical populations. II. The age structure of T. rubescens. Rev Biol Trop. 1979;27: 293–300.

[60] Matsuura Y. A probable cause of recruitment failure of Brazilian sardine, Sardinella aurita, population during the 1974/75 spawning season. South African J Mar Sci. 1996;17: 29–35.

[61] Sazima I. Similarities in feeding behaviour between some marine and freshwater fishes of two tropical communities. J Fish Biol. 1986;29: 53–65.

[62] Vasconcelos-Filho A, Neumann-Leitão S, Eskinazi-Leça E, Oliveira A, Porto-Neto F. Hábitos alimentares de consumidores primários da ictiofauna do sistema estuarino de Itamaracá (Pernambuco—Brasil). Rev Bras Eng Pesca. 2009;4: 21–31.

[63] Rueda PS. Stomach content analysis of Mugil cephalus and Mugil curema (Mugiliformes: Mugilidae) with emphasis on diatoms in the Tamiahua lagoon, México. Rev Biol Trop. 2002;50: 245–252. 12298252

[64] Isangedighi IA, Udo PJ, Ekpo IE. Diet composition of Mugil cephalus (Pisces: Mugilidae) in the cross river estuary, Niger delta, Nigeria. J Agric Food Environ. 2009;5: 10–15.

[65] Modou SS, Mouhameth C, Tinkoudgou KJA. Seasonal feeding variation of the yellow mule (Mugil cephalus, Linnaeus 1758, Mugilidae) in Senegal River estuary fishery. Int J Agric Policy Res. 2014;2: 125–131.

[66] Dallagnolo R, Schwingel PR, Perez JAA. Estimativas de Produção Anual de Sardinha-verdadeira (Sardinella brasiliensis) em Santa Catarina: um modelo de projeção de capturas a partir dos padrões mensais de desembarque no Estado. Brazilian J Aquat Sci Technol. 2010;14: 95–104.

[67] Jablonski S, Legey L. Quantifying environmental effects on the recruitment of Brazilian sardine (Sardinella brasiliensis), 1977–1993. Sci Mar. 2004;68: 385–398.

[68] Gigliotti ES, Gherardi DFM, Paes ET, Souza RB, Katsuragawa M. Spatial analysis of egg distribution and geographic changes in the spawning habitat of the Brazilian sardine Sardinella brasiliensis. J Fish Biol. 2010;77: 2248–2267. doi: 10.1111/j.1095-8649.2010.02802.x 21155781 10.1111/j.1095-8649.2010.02802.x

[69] Azam F, Fenchel T, Field JG, Meyer-Reil LA, Thingstad F. The ecological role of water column microbes in the sea. Mar Ecol Prog Ser. 1983;10: 257–263.

[70] Azam F, Malfatti F. Microbial structuring of marine ecosystems. Nat Rev Microbiol. 2007;5: 782–791. doi: 10.1038/nrmicro1747 17853906 10.1038/nrmicro1747

[71] Thompson SA, Sydeman WJ, Santora JA, Black BA, Suryan RM, Calambokidis J, et al Linking predators to seasonality of upwelling: Using food web indicators and path analysis to infer trophic connections. Prog Oceanogr. Elsevier Ltd; 2012;101: 106–120. doi: 10.1016/j.pocean.2012.02.001

[72] Sommer U, Stibor H. Copepoda–Cladocera–Tunicata: The role of three major mesozooplankton groups in pelagic food webs. J Plankton Res. 2002;17: 161–174.

[73] Haimovici M, Perez JAA. Abundância e distribuição de cefalópodes em cruzeiros de prospecção pesqueira demersal na plataforma externa e talude continental do sul do Brasil. Atlântica. 1991;13: 189–200.

[74] Bakun A, Parrish RH. Comparative studies of coastal pelagic fish reproductive habitats: the anchovy (Engraulis anchoita) of the southwestern Atlantic. ICES J Mar Sci. 1991;48: 343–361.

[75] Valentin JL. The Cabo Frio upwelling system, Brazil In: Seeliger U, Kjerfve B, editors. Coastal marine ecosystems of Latin America. Berlin: Springer-Verlag; 2001 pp. 97–105.

