# Supplementary material for: Inter-annual cascade effect on marine food web: A benthic pathway lagging nutrient supply to pelagic fish stock
Source: PLoS One. 2017 Sep 8;12(9):e0184512. doi: 10.1371/journal.pone.0184512 (PMC5590966; doi:10.1371/journal.pone.0184512)
Supplement: S1 Table — (DOCX) [file pone.0184512.s001.docx]

**S1 Table. Monthly anomalies of SST.**

|  | **1995** | **1996** | **1997** | **1998** | **1999** | **2000** | **2001** | **2002** | **2003** | **2004** | **2005** | **2006** | **2007** | **2008** | **2009** |
| --- | --- | --- | --- | --- | --- | --- | --- | --- | --- | --- | --- | --- | --- | --- | --- |
| **Jan** | 1.12 | 0.80 | -1.29 | 0.64 | 1.82 | -1.01 | 0.39 | -0.06 | 0.21 | 0.38 | -0.76 | -0.12 | -1.76 | -1.06 | 0.70 |
| **Feb** | 1.87 | 1.30 | -2.07 | 0.12 | 0.03 | -0.66 | 0.76 | 1.01 | -0.80 | -0.93 | -0.24 | 0.16 | -0.84 | -0.13 | 0.40 |
| **Mar** | 1.31 | 1.58 | -0.31 | -0.37 | -0.31 | -1.64 | -0.36 | -1.43 | -0.36 | 0.33 | 1.06 | 1.14 | -1.17 | -0.21 | 0.72 |
| **Apr** | -1.44 | 0.34 | -0.90 | 0.72 | -0.28 | 0.12 | -0.55 | -1.38 | 1.80 | -0.01 | -1.29 | 0.63 | 1.60 | 0.09 | 0.54 |
| **May** | 0.85 | -0.64 | -2.45 | 0.46 | -0.28 | -0.60 | 0.33 | -0.18 | 1.76 | -0.77 | 0.93 | 1.11 | -0.48 | 0.13 | -0.17 |
| **Jun** | 0.39 | -0.49 | -2.15 | 1.08 | -0.65 | -1.32 | 1.00 | 1.12 | 1.66 | -0.13 | -0.73 | 0.25 | 0.20 | -0.16 | -0.08 |
| **Jul** | 1.62 | -0.67 | -0.45 | 0.52 | -0.26 | -2.45 | 0.79 | 0.84 | 1.32 | 0.09 | -0.75 | -0.11 | 0.48 | -0.55 | -0.42 |
| **Aug** | 1.57 | -0.97 | -0.71 | 1.35 | -0.95 | -1.28 | 0.02 | 0.65 | 1.14 | -1.76 | 0.75 | 0.20 | -0.02 | 0.40 | -0.39 |
| **Sep** | 1.09 | -1.63 | -0.23 | 1.01 | -1.41 | 0.56 | -0.85 | -0.66 | 0.90 | -0.62 | 0.72 | 1.25 | -1.18 | 0.98 | 0.10 |
| **Oct** | -0.24 | -0.80 | 0.22 | 1.45 | -1.73 | 1.13 | -1.10 | -0.27 | 0.01 | 0.50 | 1.31 | 0.89 | -1.32 | -0.76 | 0.70 |
| **Nov** | 0.19 | -1.19 | 1.53 | -0.16 | -2.69 | -0.74 | 0.72 | -0.07 | 0.72 | 0.37 | 0.84 | -0.05 | -0.37 | 0.40 | 0.47 |
| **Dec** | 0.88 | -2.19 | 0.15 | 0.66 | -1.16 | -0.45 | -0.40 | 0.41 | -0.58 | 1.02 | -1.15 | 0.69 | 0.81 | -0.17 | 1.49 |
