# Supplementary material for: Inter-annual cascade effect on marine food web: A benthic pathway lagging nutrient supply to pelagic fish stock
Source: PLoS One. 2017 Sep 8;12(9):e0184512. doi: 10.1371/journal.pone.0184512 (PMC5590966; doi:10.1371/journal.pone.0184512)
Supplement: S2 Table — (DOCX) [file pone.0184512.s002.docx]

**S2 Table. Monthly anomalies of NO2+NO3.**

|  | **1995** | **1996** | **1997** | **1998** | **1999** | **2000** | **2001** | **2002** | **2003** | **2004** | **2005** | **2006** | **2007** | **2008** | **2009** |
| --- | --- | --- | --- | --- | --- | --- | --- | --- | --- | --- | --- | --- | --- | --- | --- |
| **Jan** | 0.37 | -0.89 | 0.71 | -0.50 | -1.33 | 0.66 | -0.14 | -0.02 | 0.33 | -0.55 | -0.48 | -1.08 | 0.32 | 2.85 | -0.25 |
| **Feb** | -0.94 | -0.68 | 0.01 | -1.17 | -0.94 | 0.07 | 0.38 | 0.43 | 2.75 | 0.21 | -0.38 | -0.68 | -0.68 | 0.99 | 0.64 |
| **Mar** | -0.63 | -0.60 | 0.02 | -0.23 | -0.48 | 0.17 | -0.29 | 0.20 | -0.33 | -0.37 | -0.90 | -0.53 | -0.55 | 1.59 | 2.94 |
| **Apr** | 2.19 | -0.36 | -0.51 | -0.45 | -0.78 | -0.47 | -0.37 | 1.16 | -0.43 | -0.48 | 2.19 | -0.64 | -0.54 | -0.65 | 0.13 |
| **May** | -0.97 | -0.66 | -0.86 | -0.23 | -0.90 | -0.51 | 1.43 | -0.08 | 1.19 | -0.59 | -0.10 | -0.72 | -0.53 | 1.77 | 1.74 |
| **Jun** | -1.05 | -0.24 | 0.02 | -1.03 | 0.69 | 0.70 | -0.15 | -0.05 | 0.31 | -0.87 | -0.84 | -1.29 | 0.56 | 2.62 | 0.61 |
| **Jul** | -1.22 | -0.08 | -1.03 | 0.83 | -0.43 | 1.15 | 0.30 | -0.33 | -0.12 | -0.44 | -0.42 | 2.86 | -0.60 | -0.26 | -0.21 |
| **Aug** | -1.17 | -0.72 | 0.83 | -0.65 | 1.16 | -0.07 | 0.63 | -0.96 | -0.26 | 0.29 | -0.36 | 2.59 | -1.01 | -0.52 | 0.22 |
| **Sep** | -0.72 | -0.58 | -0.58 | -0.48 | 0.28 | 0.50 | 3.34 | -0.09 | -0.15 | -0.67 | 0.22 | -0.34 | -0.63 | -0.16 | 0.07 |
| **Oct** | -1.08 | -0.95 | -0.34 | -0.47 | 1.12 | 0.20 | 2.51 | -0.04 | -0.35 | -0.28 | -1.12 | -0.08 | 1.24 | -0.87 | 0.50 |
| **Nov** | -0.56 | -0.59 | -0.45 | -0.77 | 3.31 | 0.29 | -0.42 | 0.65 | 0.00 | -0.53 | -0.67 | -0.32 | -0.05 | 0.27 | -0.15 |
| **Dec** | -0.69 | 1.26 | -0.92 | -1.19 | 0.38 | 0.22 | 1.13 | 1.92 | -0.37 | -1.02 | 0.55 | -1.10 | 0.01 | 0.87 | -1.05 |
