# Supplementary material for: Inter-annual cascade effect on marine food web: A benthic pathway lagging nutrient supply to pelagic fish stock
Source: PLoS One. 2017 Sep 8;12(9):e0184512. doi: 10.1371/journal.pone.0184512 (PMC5590966; doi:10.1371/journal.pone.0184512)
Supplement: S3 Table — (DOCX) [file pone.0184512.s003.docx]

**S3 Table. Monthly anomalies of Phosphate.**

|  | **1995** | **1996** | **1997** | **1998** | **1999** | **2000** | **2001** | **2002** | **2003** | **2004** | **2005** | **2006** | **2007** | **2008** | **2009** |
| --- | --- | --- | --- | --- | --- | --- | --- | --- | --- | --- | --- | --- | --- | --- | --- |
| **Jan** | 0.75 | 0.98 | 0.88 | -1.27 | -1.64 | -0.36 | 0.30 | 1.50 | -0.03 | -0.82 | -0.72 | -0.75 | -0.06 | -0.49 | 1.72 |
| **Feb** | -0.45 | 1.16 | 0.44 | -0.68 | -1.41 | -0.96 | 0.85 | 1.61 | 0.87 | 0.82 | -0.81 | -0.98 | 0.83 | 0.05 | -1.35 |
| **Mar** | -0.02 | -0.67 | 0.29 | -0.79 | -0.81 | -0.53 | 0.63 | 2.89 | 0.51 | -0.80 | -1.18 | -0.54 | 0.05 | 0.30 | 0.69 |
| **Apr** | 1.35 | 0.33 | 0.98 | 0.61 | -0.77 | -0.88 | -0.60 | 1.86 | -0.18 | -0.66 | 0.83 | -0.74 | -1.09 | -1.55 | 0.51 |
| **May** | -0.35 | -0.18 | 0.20 | -0.98 | -1.14 | -0.60 | 1.81 | 2.48 | 0.39 | -0.72 | 0.33 | -0.16 | 0.22 | -0.46 | -0.83 |
| **Jun** | -0.13 | 0.90 | 2.30 | -1.05 | -0.13 | -0.27 | 0.60 | 0.20 | -0.01 | -0.46 | -0.56 | -0.56 | 0.46 | 0.84 | -2.13 |
| **Jul** | 0.50 | 1.36 | -0.72 | -0.83 | -0.89 | 1.06 | 1.74 | 1.00 | 0.22 | -0.70 | -0.81 | 0.63 | -0.13 | -1.56 | -0.85 |
| **Aug** | 1.85 | 0.44 | -0.46 | -0.57 | -0.26 | 0.42 | 1.66 | -0.05 | 0.59 | -0.22 | -0.54 | -0.23 | 0.59 | -2.16 | -1.06 |
| **Sep** | 0.29 | -0.30 | -0.55 | -0.54 | -0.49 | 0.13 | 0.09 | 3.44 | 0.09 | -0.63 | -0.06 | -0.06 | -0.34 | -0.34 | -0.72 |
| **Oct** | 0.62 | -0.47 | 0.12 | -0.36 | -0.77 | 0.54 | 0.84 | 2.42 | -0.78 | 0.62 | 0.17 | -1.14 | -0.71 | 0.54 | -1.64 |
| **Nov** | 0.60 | 0.98 | 0.06 | -1.22 | 0.93 | 1.02 | -0.56 | 1.96 | -0.24 | -0.65 | 0.23 | -0.60 | -0.50 | -0.02 | -1.99 |
| **Dec** | 2.46 | 1.58 | -1.38 | -1.19 | -0.41 | 0.37 | 0.37 | -0.49 | -0.02 | -0.59 | 0.66 | -0.40 | -0.23 | -0.63 | -0.08 |
