# Supplementary material for: Inter-annual cascade effect on marine food web: A benthic pathway lagging nutrient supply to pelagic fish stock
Source: PLoS One. 2017 Sep 8;12(9):e0184512. doi: 10.1371/journal.pone.0184512 (PMC5590966; doi:10.1371/journal.pone.0184512)
Supplement: S4 Table — (DOCX) [file pone.0184512.s004.docx]

**S4 Table. Monthly anomalies of Chlorophyll a.**

|  | 1995 | 1996 | 1997 | 1998 | 1999 | 2000 | 2001 | 2002 | 2003 | 2004 | 2005 | 2006 | 2007 | 2008 | 2009 |
| --- | --- | --- | --- | --- | --- | --- | --- | --- | --- | --- | --- | --- | --- | --- | --- |
| **Jan** | 0.11 | 0.08 | 0.66 | -0.49 | -1.22 | 2.19 | 0.87 | -1.56 | -0.91 | -0.36 | 0.69 | -0.68 | 0.83 | -0.87 | 0.67 |
| **Feb** | -0.11 | -0.88 | 0.79 | 1.02 | -0.35 | -0.16 | -1.03 | -0.33 | 0.06 | -0.34 | 0.08 | -0.31 | 2.99 | -0.60 | -0.83 |
| **Mar** | 0.42 | -0.38 | -0.59 | 1.20 | -0.47 | 0.08 | -1.46 | -0.06 | -0.41 | 0.47 | -0.10 | 2.77 | 0.14 | -0.54 | -1.08 |
| **Apr** | -0.28 | 0.03 | 1.04 | -0.46 | -1.14 | -0.33 | -1.84 | 0.20 | 0.05 | 2.63 | -0.29 | 0.48 | -0.33 | -0.33 | 0.58 |
| **May** | -0.12 | -0.35 | 0.29 | -0.15 | -0.27 | -0.41 | -0.79 | -0.16 | -0.30 | 0.63 | -0.15 | -0.02 | 3.34 | -0.66 | -0.87 |
| **Jun** | 0.24 | 0.52 | 0.20 | 0.56 | -0.15 | 1.52 | -2.04 | -0.35 | -0.64 | 0.64 | 1.32 | 0.86 | -0.08 | -1.37 | -1.22 |
| **Jul** | 1.10 | -0.63 | -0.40 | -0.45 | -0.21 | 0.53 | -1.75 | 0.46 | 0.14 | -0.32 | 2.78 | -0.36 | -0.55 | 0.07 | -0.42 |
| **Aug** | -0.23 | 0.97 | -0.37 | -0.37 | 1.94 | 0.16 | -0.98 | -1.42 | 0.34 | -0.03 | -0.38 | -1.11 | -0.83 | 0.42 | 1.89 |
| **Sep** | 0.25 | 2.96 | -0.52 | -0.68 | 1.13 | -0.15 | -0.35 | -0.03 | -0.83 | -0.12 | 0.10 | 0.63 | -0.99 | -0.68 | -0.70 |
| **Oct** | -0.18 | 1.78 | 1.63 | 1.90 | -0.83 | -0.93 | -1.38 | -0.16 | -0.39 | -0.37 | -0.69 | 0.16 | 0.11 | -0.24 | -0.40 |
| **Nov** | -0.27 | 1.38 | -0.33 | -0.18 | -0.18 | -0.31 | -1.54 | -0.31 | -0.21 | 1.97 | -0.35 | -0.43 | 1.97 | -1.08 | -0.12 |
| **Dec** | -0.03 | -0.08 | -0.93 | -0.44 | 1.43 | 0.10 | -1.51 | -1.21 | 0.00 | 1.23 | 1.57 | -0.56 | 0.41 | -1.14 | 1.18 |
