# Supplementary material for: Inter-annual cascade effect on marine food web: A benthic pathway lagging nutrient supply to pelagic fish stock
Source: PLoS One. 2017 Sep 8;12(9):e0184512. doi: 10.1371/journal.pone.0184512 (PMC5590966; doi:10.1371/journal.pone.0184512)
Supplement: S5 Table — (DOCX) [file pone.0184512.s005.docx]

**S5 Table. Monthly anomalies of Barnacle Larvae (Exclusively nauplii).**

|  | **1995** | **1996** | **1997** | **1998** | **1999** | **2000** | **2001** | **2002** | **2003** | **2004** | **2005** | **2006** | **2007** | **2008** | **2009** |
| --- | --- | --- | --- | --- | --- | --- | --- | --- | --- | --- | --- | --- | --- | --- | --- |
| **Jan** | -0.68 | -0.69 | -0.59 | -0.40 | 1.08 | 0.30 | -0.63 | 2.95 | -0.04 | 1.01 | -0.35 | -0.58 | -0.52 | -0.27 | -0.61 |
| **Feb** | -0.53 | -0.74 | -0.81 | -0.61 | -0.53 | 0.97 | -0.44 | 0.54 | -0.19 | 0.70 | 0.53 | 2.91 | -0.43 | -0.77 | -0.60 |
| **Mar** | -0.04 | 0.73 | -0.96 | -0.31 | -0.69 | -0.59 | 2.79 | -0.25 | -0.13 | -0.06 | -0.69 | 0.74 | 1.15 | -0.75 | -0.92 |
| **Apr** | 0.15 | 0.81 | -1.33 | 0.36 | -0.45 | -0.35 | -0.43 | -0.10 | 1.08 | 2.56 | -0.69 | 0.74 | -0.33 | -1.09 | -0.94 |
| **May** | -0.59 | -0.76 | -0.84 | -0.65 | 0.09 | -0.53 | -0.35 | -0.44 | 0.62 | 1.84 | -0.41 | 2.33 | -0.69 | 1.04 | -0.65 |
| **Jun** | -0.73 | 0.07 | -0.81 | -0.43 | -0.55 | -0.34 | 0.08 | 2.75 | -0.39 | 1.92 | -0.15 | -0.10 | -0.52 | -0.11 | -0.70 |
| **Jul** | -0.49 | -0.42 | -0.63 | -0.49 | -0.41 | 0.13 | -0.22 | 3.38 | -0.30 | -0.29 | 0.85 | -0.05 | -0.49 | -0.39 | -0.16 |
| **Aug** | -0.33 | -0.73 | -0.90 | -0.24 | 3.12 | -0.36 | 0.31 | 0.87 | -0.50 | -0.46 | -0.09 | -0.23 | -0.81 | -0.35 | 0.70 |
| **Sep** | -0.93 | -0.99 | -1.25 | 0.60 | 0.29 | -0.44 | 2.35 | 0.85 | 0.37 | 1.43 | -0.54 | -0.94 | -0.57 | -0.09 | -0.12 |
| **Oct** | -0.28 | -1.05 | -1.36 | -0.59 | 0.99 | -0.81 | 2.28 | -0.26 | 0.94 | 0.84 | -0.45 | -0.34 | -0.87 | 1.04 | -0.08 |
| **Nov** | -0.70 | -0.91 | -1.23 | 1.72 | 0.71 | -0.14 | 1.27 | -0.67 | -0.67 | 1.29 | 0.29 | -1.12 | 0.01 | -0.95 | 1.11 |
| **Dec** | -0.25 | -1.19 | 0.87 | 2.24 | 0.54 | 1.46 | 0.35 | -0.52 | -0.35 | -0.23 | -1.22 | 0.63 | -0.76 | -0.93 | -0.66 |
