# Supplementary material for: Inter-annual cascade effect on marine food web: A benthic pathway lagging nutrient supply to pelagic fish stock
Source: PLoS One. 2017 Sep 8;12(9):e0184512. doi: 10.1371/journal.pone.0184512 (PMC5590966; doi:10.1371/journal.pone.0184512)
Supplement: S6 Table — (DOCX) [file pone.0184512.s006.docx]

**S6 Table. Monthly anomalies of Mussel Larvae (Exclusively Mytilidae).**

|  | **1995** | **1996** | **1997** | **1998** | **1999** | **2000** | **2001** | **2002** | **2003** | **2004** | **2005** | **2006** | **2007** | **2008** | **2009** |
| --- | --- | --- | --- | --- | --- | --- | --- | --- | --- | --- | --- | --- | --- | --- | --- |
| **Jan** | -0.49 | -0.45 | -0.59 | -0.48 | 0.59 | -0.32 | 0.20 | 3.03 | -0.05 | 0.16 | 0.17 | 0.92 | -0.99 | -0.95 | -0.73 |
| **Feb** | -0.05 | -0.77 | -0.85 | -0.64 | -0.44 | -0.09 | 0.12 | 1.13 | 2.45 | 0.15 | -0.69 | 1.65 | -0.96 | -0.72 | -0.27 |
| **Mar** | 1.31 | 0.15 | -0.84 | -0.63 | -0.40 | -0.69 | 2.88 | -0.43 | 0.77 | 0.07 | -0.04 | -0.24 | -0.80 | -0.35 | -0.79 |
| **Apr** | -0.19 | 0.71 | -0.74 | -0.65 | -0.36 | -0.48 | 0.01 | -0.53 | 3.14 | -0.09 | -0.59 | 0.94 | -0.62 | 0.08 | -0.62 |
| **May** | -0.41 | -0.67 | -0.85 | 0.36 | -0.68 | 0.14 | 0.27 | -0.52 | 2.71 | 1.61 | 0.27 | -0.06 | -0.81 | -0.42 | -0.94 |
| **Jun** | -0.82 | -0.52 | -1.11 | -0.14 | 1.59 | 1.71 | 0.56 | 0.43 | -0.23 | 1.68 | -0.98 | -0.28 | -1.25 | -0.03 | -0.61 |
| **Jul** | -0.15 | -0.83 | -0.85 | 0.42 | 0.04 | -0.82 | -0.29 | 2.19 | 2.00 | -0.45 | 0.46 | 0.33 | -1.22 | -0.91 | 0.07 |
| **Aug** | 1.36 | -0.95 | -0.54 | 0.45 | 2.15 | 0.64 | 0.15 | 1.14 | -0.45 | -0.68 | 0.41 | -0.83 | -1.27 | -0.70 | -0.87 |
| **Sep** | 2.17 | -0.63 | -1.05 | -0.65 | 0.25 | 0.25 | 0.07 | -0.08 | 1.87 | 0.20 | 0.63 | -0.37 | -1.51 | -0.91 | -0.24 |
| **Oct** | 1.04 | -0.88 | -1.18 | -0.30 | 1.99 | 0.24 | 1.96 | 0.27 | -0.40 | -0.33 | 0.46 | -0.68 | -1.01 | -0.77 | -0.42 |
| **Nov** | 1.76 | -0.42 | -0.58 | 0.02 | -0.47 | 2.98 | -0.27 | -0.42 | -0.44 | -0.19 | -0.31 | -0.33 | -0.56 | -0.40 | -0.38 |
| **Dec** | 0.21 | -0.55 | 0.32 | 0.59 | -0.57 | 3.35 | 0.17 | -0.30 | -0.40 | -0.47 | -0.57 | -0.24 | -0.62 | -0.48 | -0.44 |
