# Supplementary material for: Inter-annual cascade effect on marine food web: A benthic pathway lagging nutrient supply to pelagic fish stock
Source: PLoS One. 2017 Sep 8;12(9):e0184512. doi: 10.1371/journal.pone.0184512 (PMC5590966; doi:10.1371/journal.pone.0184512)
Supplement: S7 Table — (DOCX) [file pone.0184512.s007.docx]

**S7 Table. Monthly anomalies of Fish.**

|  | **1995** | **1996** | **1997** | **1998** | **1999** | **2000** | **2001** | **2002** |
| --- | --- | --- | --- | --- | --- | --- | --- | --- |
| **Jan** | -0.60 | 1.00 | 1.37 | 0.81 | -0.04 | -0.60 | -1.63 | -0.32 |
| **Feb** | -0.10 | 1.84 | 0.83 | 0.11 | -0.86 | -0.23 | -0.14 | -1.45 |
| **Mar** | 2.40 | 0.11 | -0.64 | -0.41 | -0.14 | -0.62 | -0.26 | -0.44 |
| **Apr** | 2.35 | 0.20 | -0.39 | -0.60 | -0.28 | -0.19 | -0.23 | -0.86 |
| **May** | 0.76 | 2.11 | -1.09 | -0.12 | -0.62 | -0.35 | -0.49 | -0.20 |
| **Jun** | -0.43 | 2.44 | -0.35 | -0.28 | -0.60 | -0.49 | -0.07 | -0.22 |
| **Jul** | -0.81 | 1.14 | -0.64 | -0.20 | 0.08 | 0.59 | 1.37 | -1.54 |
| **Aug** | -0.64 | 2.33 | -0.94 | -0.02 | -0.19 | -0.02 | -0.06 | -0.47 |
| **Sep** | 0.93 | 2.05 | -0.82 | -0.50 | -0.20 | -0.16 | -0.41 | -0.89 |
| **Oct** | 2.24 | 0.15 | -0.78 | 0.18 | -0.12 | -0.04 | -0.83 | -0.80 |
| **Nov** | 2.24 | 0.51 | -0.39 | -0.58 | -0.62 | 0.05 | -0.37 | -0.83 |
| **Dec** | 0.89 | 1.99 | -0.89 | -0.39 | 0.18 | -0.32 | -0.50 | -0.96 |
