# Supplementary material for: Inter-annual cascade effect on marine food web: A benthic pathway lagging nutrient supply to pelagic fish stock
Source: PLoS One. 2017 Sep 8;12(9):e0184512. doi: 10.1371/journal.pone.0184512 (PMC5590966; doi:10.1371/journal.pone.0184512)
Supplement: S8 Table — (DOCX) [file pone.0184512.s008.docx]

**S8 Table. Annual anomalies of Fish.**

|  | **Total Catch** | ***Sardinella brasiliensis*** | ***Mugil liza*** | ***Caranx latus*** |
| --- | --- | --- | --- | --- |
| **1995** | 2.3 | 2.3 | 1.2 | -1.2 |
| **1996** | -0.4 | -0.4 | -0.3 | 1.3 |
| **1997** | 0.5 | 0.5 | -0.3 | -0.9 |
| **1998** | -0.6 | -0.7 | 1.6 | 0.3 |
| **1999** | -0.2 | -0.2 | 0.5 | 0.2 |
| **2000** | -0.4 | -0.4 | -0.8 | 1.5 |
| **2001** | -0.6 | -0.6 | -0.9 | -0.4 |
| **2002** | -0.6 | -0.5 | -1.0 | -0.7 |
