# Supplementary figures and images for: Inter-annual cascade effect on marine food web: A benthic pathway lagging nutrient supply to pelagic fish stock
Source: PLoS One. 2017 Sep 8;12(9):e0184512. doi: 10.1371/journal.pone.0184512 (PMC5590966; doi:10.1371/journal.pone.0184512)

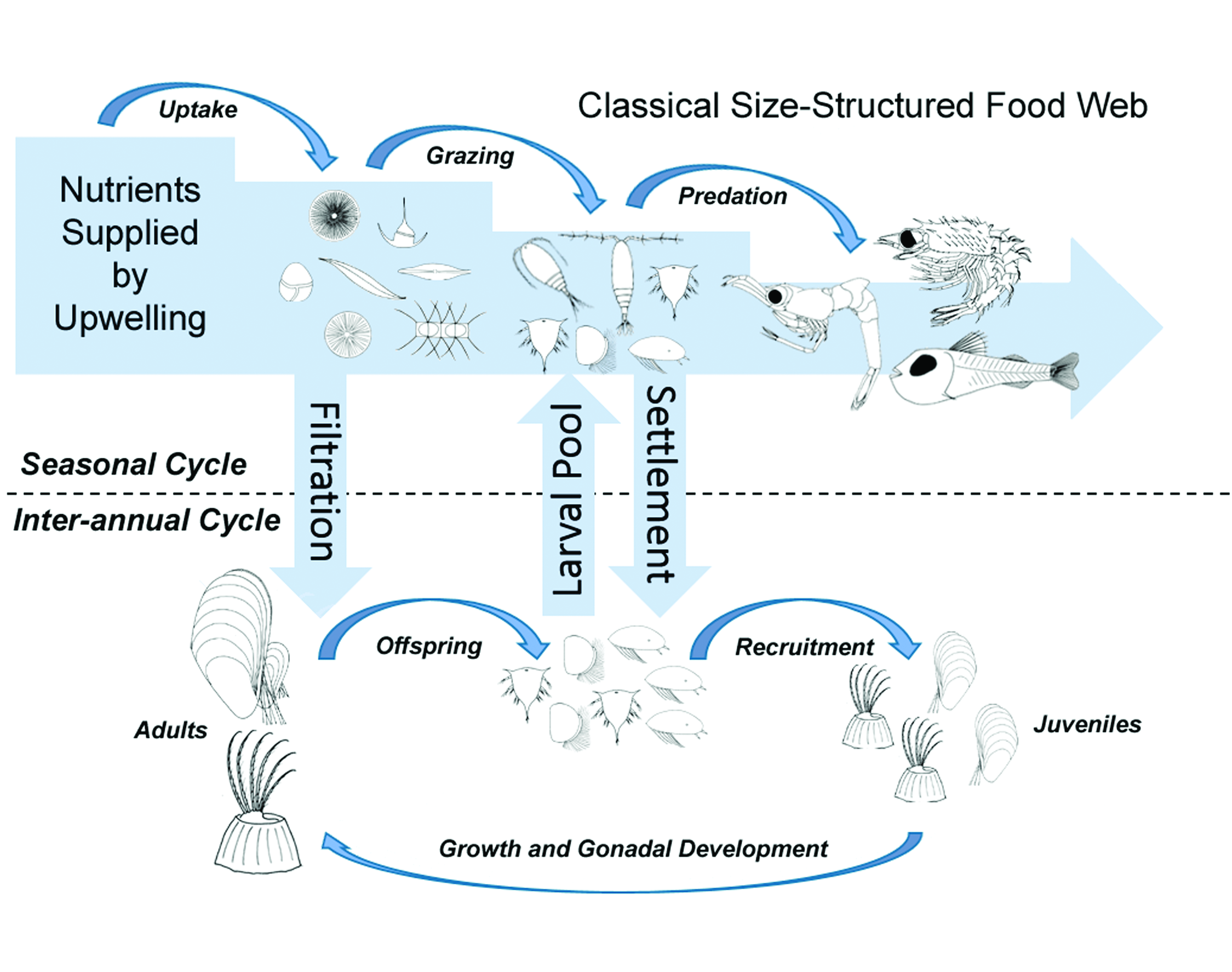

Supplement: S1 Fig — Energy and matter consumed by phytoplankton started a bottom-up stimulus that is not completely transmitted to the next trophic level in the plankton. Benthic filter-feeding organisms like barnacles and mussels can consume the majority of phytoplankton (Filtration) exerting a competitive top-down control that will start an inter-annual cascading effect. Settlement is an additional process that can trap some energy into the ontogenetic development of benthic organisms and alter the energy flow in the planktonic food web. (TIF) [file pone.0184512.s009.tif]
